# Families Implementing Resilient Systems Together (FIRST)

**DOI:** 10.3390/children13040572

**Published:** 2026-04-20

**Authors:** Ariane Marie-Mitchell, Catherine A. Tan, Elizabeth Park, Gabriela A. Plascencia, Cameron L. Neece

**Affiliations:** 1 Department of Preventive Medicine and Pediatrics, Loma Linda University, Loma Linda, CA 92350, USA; catan@llu.edu; 2 Department of Pediatrics, Loma Linda University, Loma Linda, CA 92354, USA; gaplascencia@llu.edu; 3 Department of Pediatrics, SAC Health, San Bernardino, CA 92410, USA; 4 Department of Behavioral Health, SAC Health, San Bernardino, CA 92410, USA; elipark@llu.edu; 5 Department of Family Medicine, Loma Linda University, Loma Linda, CA 92354, USA; 6 Department of Psychology, Loma Linda University, Loma Linda, CA 92350, USA; cneece@llu.edu

**Keywords:** adverse childhood experiences, resilience, screening, pediatrics

## Abstract

**Highlights:**

**What are the main findings?**

- Results of physician training about ACEs and Resilience.
- Research protocol for the FIRST study.

**What are the implications of the main findings?**

- Approach to addressing ACEs during well-child care.
- Need for research on pediatric preventive care.

**Abstract:**

**Background/Objectives**: Prior research suggests that it is possible to improve health outcomes in children with adverse childhood experiences (ACEs) through multi-component interventions that promote protective factors. We designed the Families Implementing Resilient Systems Together (FIRST) study to address the gaps in research on the potential effectiveness of screening for specific ACEs through pediatric practice. **Methods**: As part of our clinical quality improvement efforts to improve patient care for children impacted by ACEs, we trained a random sample of pediatricians on strategies to promote protective factors and encouraged them to make referrals to community health workers (CHWs) and parenting education resources. This manuscript describes our clinic data on practice changes associated with the FIRST physician training, and our data collection plan for our research study. **Results**: Physician training resulted in attitudinal shifts and measurable behavioral changes. Trained providers made referrals to CHWs for approximately 5–10% of well-child care visits. The majority (84%) of referrals were for multiple risk factors, most commonly ACEs and socioeconomic concerns. The most common ACEs were parental divorce/separation, parent–child verbal abuse, and caregiver mental health problems. **Conclusions**: FIRST training improves counseling, education and referrals for children exposed to ACEs. Our research study will evaluate the impact of the FIRST intervention and address important questions about associations between specific ACEs, protective factors, and biomarkers of toxic stress.

## 1. Introduction

The science of adverse childhood experiences (ACEs) starts with understanding the biology of stress. When we are stressed, there is a disconnect between the prefrontal cortex and limbic system or the thinking and feeling parts of our brain. There is also an activation of the sympathetic nervous system which affects our entire body down to our blood cells and immune system [1]. If the stress response is activated briefly, such as when you need to run away from a tiger, that can save your life. However, if the stress response is activated repeatedly, day after day after day, that can wear away at both mind and body [2]. This is what can happen with ACEs [3].

The first study published in the medical literature that used the term “ACEs” defined these as experiences prior to the age of 18 of child abuse, child neglect or household dysfunction including caregiver incarceration, mental illness, substance abuse, domestic violence, and divorce or separation [4]. Notice that this definition describes family-level risk factors that directly affect the home environment and parent–child relationships. More recent studies have used broader definitions of ACEs that include socioeconomic stressors and discrimination [5]. ACEs increase risk of mental health disorders, chronic medical conditions, academic difficulties and negative social outcomes [6,7]. Socioeconomic stressors and discrimination increase risk of similar poor outcomes [8,9,10]. This is not surprising since different types of psychosocial risk factors activate the same physiological stress response.

**Figure 1:**
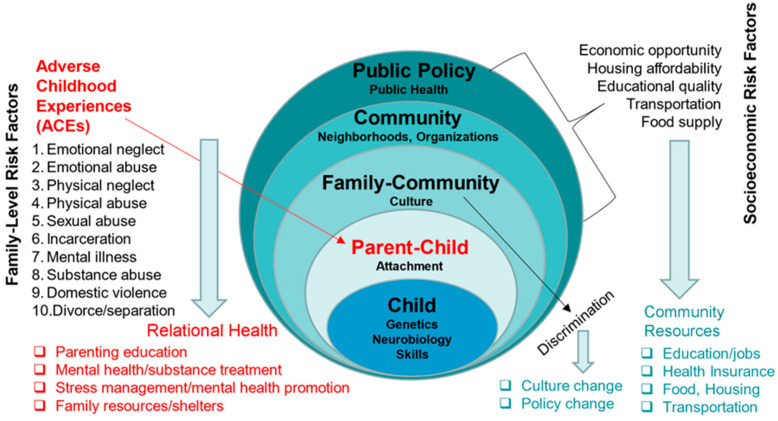
Ecological model of the developing child.

Nonetheless, when designing a psychosocial intervention to successfully reduce the impact of ACEs and promote healthy child development, it is important to distinguish between family-level and other types of stressors. As the ecological model of the developing child illustrates (Figure 1), a premise of the Families Implementing Resilient Systems Together (FIRST) intervention is that family-level risk factors require interventions that focus on promoting relational health. By contrast, socioeconomic stressors and discrimination require other types of interventions. While individuals are at highest risk when they experience multiple risk factors [11,12], it is overly simplistic to lump all risk factors or to frame socioeconomic stressors as the “root causes” of ACEs [13]. It is important to remember that one of the most consequential findings of the original ACE research is that ACEs occur across all socioeconomic strata and, therefore, are a distinct, influential cluster of risk factors that require interventions that focus on promoting healthy family relationships [4].

### 1.1. “Toxic Stress” as a Mediator of Poor Health Outcomes

The term “toxic stress” has become a popular way to explain the association between severe or chronic stressors, such as ACEs, and poor health outcomes [14]. The idea is that chronic stress activation can cause wear and tear on the body and that over time this becomes sufficiently “toxic” to result in pathology. Different individuals may develop different types of pathology depending upon other genetic or environmental factors, but toxic stress is the common mechanism. A substantial body of human and animal research supports this idea and generally confirms that early life stressors tend to create environments that are detrimental for healthy development [15,16].

When considering the natural history of disease, toxic stress can be thought of as a pre-clinical condition between risk factors (such as ACEs) and the onset of clinical conditions (such as poor health outcomes associated with ACEs). With this framework in mind, it would be ideal to have a clearly defined method to distinguish individuals who have and do not have toxic stress. Such a method could then be used to assess the impact of actions taken to prevent the accumulation of ACEs or the development of disease in a pediatric patient. A few studies have examined biomarkers of toxic stress in pediatric populations [17], but to date there is no gold standard method to measure toxic stress. Thus, research is needed to better understand how to translate basic science research on early life stress into patient metrics of toxic stress.

### 1.2. Adding Protective Factors to the Equation

ACEs and resilience are two sides of the same coin, where resilience can be defined as our ability to “bounce back” from adversity [18]. Just as in real life you cannot have one without the other (it is adversity that generates the need to bounce back), so too in health care practice is it critical to recognize that the reason that we screen for specific ACEs is so that we can provide counseling and connections to resources which will build resilience [19]. So-called “positive childhood experiences” (PCEs) are a way of referring to factors that we know protect health and enable the “bounce back” [20,21].

Prior research suggests that it is possible to improve health outcomes in children with ACEs through multi-component interventions that promote protective factors described by the Strengthening Families Framework as concrete support, parental resilience, secure attachments, knowledge of parenting and child development, and social connections [22,23]. There is also growing evidence that interventions targeting one or more protective factors can reduce physiological measures of stress [24,25,26]. Thus, there is good reason to believe that pediatric interventions that factor in what is known about protective factors may be better able to understand individual variability in response to ACEs, to identify biomarkers of toxic stress, and to successfully support families.

### 1.3. Evaluating the Effectiveness of Screening for Specific ACEs

**Figure 2:**
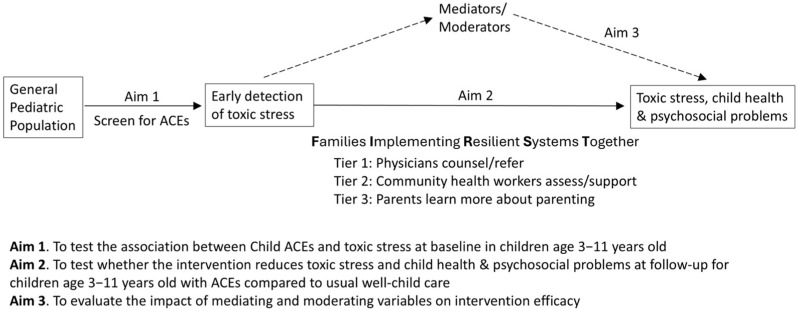
Analytic framework for FIRST research study.

We designed the FIRST study to address the gaps in research on the effectiveness of screening for specific ACEs through pediatric practice. Our overall objective was to determine whether pediatrician counseling and interventions for ACEs can either reduce toxic stress in children and/or improve child outcomes. To address this overall objective, our research study incorporated critical questions about associations between child ACEs and potentially feasible clinical metrics of toxic stress, as well as questions about whether the FIRST intervention successfully reduces toxic stress and child health and psychosocial problems. The analytic framework for our research study is shown in Figure 2.

The FIRST intervention was designed to be integral to well-child care rather than something extra to add to pediatric practice. In other words, the FIRST intervention was a specific approach to conducting well-child care that integrated counseling and intervention for ACEs alongside other recommended well-child care activities. We do not know of any other interventions to address child ACEs that have taken this approach. In addition, the FIRST intervention advanced beyond existing pediatric interventions by systematically training pediatricians to address multiple ACE exposures and risk of ACEs. At the same time, the FIRST intervention intentionally mirrored other successful pediatric interventions, such as the Safe Environment for Every Kid (SEEK) model [27], by providing handouts about protective factors, as well as connections to community resources when needed.

We were in the unique position to be able to conduct the FIRST study because our pediatric clinics were engaged in clinical quality improvement work related to ACEs. In particular, all of our pediatricians were already screening for specific ACEs as part of routine well-child care visits. In order to improve the effectiveness of this screening, we developed the FIRST intervention and randomly assigned pediatricians to training on strategies to promote protective factors. We also encouraged trained providers to make referrals to community health workers (CHWs) and parenting education resources. We nested the FIRST research study within the context of this clinical quality improvement work. We recruited three cohorts from patients who had recent well-child care visits: patients with no ACEs, patients with ACEs who received usual well-child care, and patients with ACEs who received well-child care from one of our FIRST-trained pediatricians. At research visits, we collected more extensive data on ACEs, protective factors, biomarkers of stress, and child health outcomes. This manuscript describes our clinical quality improvement involving the FIRST intervention and our research protocol.

## 2. Materials and Methods

### 2.1. Clinical Quality Improvement

- Random assignment to FIRST training

Pediatric interns and faculty were randomly assigned to participate in the FIRST curriculum based upon clinic schedules. This approach to assignment was random because (1) there was no plausible mechanism to systematically associate physician clinic schedule and our primary research study outcomes (child biomarkers, health and psychosocial problems); and (2) there was no opportunity for self-selection since training assignments were not selected by physicians. In addition, clinic schedulers and patients were blind to physician training status, so whether patients saw a trained or untrained provider was random.

- FIRST training and handouts

The FIRST curriculum was designed to empower professionals with the knowledge and skills needed to strengthen families and build resilience in youth by: (1) understanding trauma and promoting resilience [14,19]; (2) supporting stress management and executive function through mindfulness, emotional self-regulation, and healthy lifestyle [28,29,30,31,32]; and (3) creating safe, stable, nurturing relationships to encourage social–emotional development [33,34,35,36,37]. The curriculum also included practicing motivational interviewing skills and making referrals to CHWs and parenting programs. Key content from the training was summarized in handouts that were made available to families and included related community resources [38].

All CHWs had prior didactic and practical training in home visitation, behavioral health, and strategies for identifying and solving problems pertaining to social determinants of health. This prior training was augmented by FIRST trainings which included the same content as the physician curriculum. However, rather than emphasizing screening during well-child visits, these sessions focused on the use of a semi-structured interview guide assessing family needs and goals. After the initial assessment, CHWs provided support as needed, and proactively contacted families for a follow-up assessment at 6 and 12 months.

Both physician and CHW trainings were interactive and 3–4 h in length. In addition, quarterly case-based discussions were provided throughout the academic year for clinical staff and community partners, along with periodic updates and reminders via staff meetings or emails. Also, both pediatric providers and CHWs encouraged families to participate in one of several available parenting programs.

While the FIRST curriculum was not evidence-based, it was informed by evidence that physician counseling and referrals to community resources could help address concrete needs and reduce risk for child maltreatment [39,40,41,42]. By creating linkages between primary care and CHWs, we applied knowledge from a robust body of literature that shows home visitors who provide social support, social service referrals and parenting education can decrease child maltreatment and behavior problems while improving child development and health outcomes [43,44,45,46,47]. Also, by encouraging participation in parenting education, we built upon an additional literature that demonstrates that learning about positive parenting strategies can reduce child behavior problems and enhance parent psychosocial well-being [48,49,50].

- Physician training feedback

Brief surveys were distributed immediately before and after the FIRST physician training. In addition, in March 2025 we distributed an electronic survey to residents and faculty who had completed the training to assess its perceived impact on clinical practice. We also conducted informal interviews with trained residents and faculty to solicit additional feedback.

- Physician direct observations

Physicians were observed during well-child visits between June 2021 to February 2022 and again June 2024 to July 2024. A medical student or research assistant who was blind to the training status of the provider documented topics discussed, concerns, and actions in a consecutive series of well-child care visits. ACE topics included risk of neglect, domestic violence, child physical abuse, parent substance abuse, parent mental health, parent incarceration, divorced/separated parenting, lack of family support, child verbal abuse, child sexual abuse, and corporal punishment. Non-ACE topics included bullying, gun safety, water safety, car/belt safety, child substance abuse, child mental health, sleep hygiene, physical activity, UV exposure, nutrition, and daily routines. Potential actions taken by physicians included counseling, sharing handouts on ACEs and resilience, providing community resources, referring to a CHW, and referring to behavioral health. Percentages were calculated for ACE and non-ACE topics where the numerator was how often a topic was discussed, of concern after discussion, or addressed with an action. The denominator was the total number of visits observed multiplied by the total number of topics.

- Physician documentation

Medical record reviews were conducted annually between September and May 2021–2024 to evaluate the impact of FIRST training on physician practice. Medical records were included for pediatric patients who presented for a well-child care visit. Only well-child care visits were included in our chart reviews because that is when we systematically screen patients for ACEs using the Whole Child Assessment (WCA) [38,51,52]. Medical records were excluded if no ACEs were reported during that visit because we were interested in evaluating counseling and referrals for ACEs. Medical records were included if the visit was with a trained provider up to a maximum of 50 per month. Medical records for well-child visits by untrained providers were selected consecutively and matched to the trained provider sample on child age and ACE score. To make sure that encounters by untrained providers were representative of usual well-child care, medical records were excluded if a referral for ACEs was made by an untrained provider. Charts were reviewed for documentation of counseling and handouts about ACEs. For a balancing measure, we also reviewed charts for documentation of physician counseling about lifestyle.

- Physician referrals to CHWs

Navigating referral pathways for specific patient needs can be challenging. Clinicians may have difficulty recalling the appropriate referral for a given concern and there is often overlap in the domains of ACEs, developmental concerns, and socioeconomic social drivers of health (SDOH). This fragmentation can create inefficiencies and increase the likelihood of missed or delayed connections to supportive services.

To address these barriers, a single, streamlined referral—the Whole Family Care order—was developed and directed to a centralized coordination hub. This consolidated order was designed to reduce confusion, minimize time burden, and facilitate appropriate linkage to services. Physicians could use one referral pathway to document any identified concerns within the three domains (ACEs, SDOHs and development). Families were connected to the appropriate resources based on a predetermined algorithm. Implementation of this unified referral process was associated with a significant increase in the number of referrals placed.

Between 6/1/21 and 3/31/24, the number of referrals made each month by trained providers that included concern for one or more ACEs was tracked, as was the success rate which was defined as the linkage of a family to a CHW.

- Physician referrals to parenting educators

Initially, our physicians were encouraged to use the Whole Family Care order to refer interested families to participate in the 16-week Nurturing Families curriculum offered by a local community organization [53]. Since participation rates were very low in this specific program, we shifted to a multi-prong strategy that allowed families to choose their approach to learning more about parenting including: (1) a handout about healthy relationships with additional resources; (2) online and in-person parenting workshops provided by local organizations; (3) online and in-person parenting groups offered by psychologists at one of our clinics; and (4) self-directed online learning through websites such as Triple P [54]. We were unable to track how many of our pediatric families chose to utilize one or more of these resources.

### 2.2. Research Study

- Participants

Pediatric patients were recruited to participate in the “Building Resilient Families” research study after attending a well-child care visit at one of five participating pediatric clinics. Fliers were posted at all of the clinics and research assistants called potentially eligible patients. Children were eligible if they were screened for ACEs, age 3–11 years old and living within a 45 min drive of the clinic. Potential participants were excluded if the child was born with a congenital health problem that would significantly impact health care utilization or prevent participation in regular education classes. Children were also excluded if parents participated in a multi-session parenting program in the last year, or if a sibling was already enrolled in the study. Three groups of participants were recruited: (1) children without ACEs who received usual well-child care and served as a no-exposure comparison; (2) children with ACEs who received usual well-child care (control group); and (3) children with ACEs who received well-child care by a provider trained in FIRST (intervention group).

- Power Calculation

Power was estimated to detect differences in the change over time between the treatment and control group using a mixed design ANOVA analysis. Our analysis was estimated to have a power of 0.80 or higher (depending on correlation strength between repeated measures, estimated at 0.30 or higher) to detect small (Cohen’s f = 0.1) standardized effect sizes when comparing longitudinal changes between groups (FIRST intervention N = 100; non-ACEs comparison group N = 70; control N = 70). Since latent growth curve (LGC) models will be used rather than ANOVA, the estimated power computed here can be viewed as a lower bound to detect the time by treatment interaction [55,56].

- Data Collection

Families who agreed to participate were scheduled for research visits within 2 weeks, 3 months, 6 months and 12 months after the child’s well-child visit. All research visits were at our research office or the family’s home, depending upon family preference. Research visits were conducted by Research Assistants who collected data from both the child and primary caregiver in either English or Spanish. A complete list of measures is shown in Table S1. All measures were collected at all visits and on all participants unless otherwise specified in Table S1.

Child ACEs were measured using caregiver self-report tools and at baseline a structured interview. Child biomarkers included biometrics, immune cell gene expression, inflammation, hemoglobin A1c, cortisol, telomere length, and evaluations of executive function. These measures were chosen based upon expert recommendations and theoretical mechanisms linking psychosocial stressors and biological disease. We included a range of measures that assessed potential toxic stress across neuroendocrine, immune, metabolic and genetic systems [57,58,59,60,61,62]. Our specific choice of measures was guided by evidence from research on biomarkers of trauma exposure in pediatric samples [63,64,65,66,67,68,69,70,71,72,73,74,75,76]. We intentionally selected minimally invasive approaches that would be easy to translate into routine pediatric practice if found to be significant.

In addition to child biomarkers, our primary outcomes also included measures of child health and psychosocial problems. We chose frequently used, standardized instruments to assess child development, child behavior, asthma control (for asthma patients) and school performance. We also reviewed electronic medical records for child chronic diagnoses and health utilization. To assess potential mediators of intervention efficacy, we collected data on potential mediators including program participation (e.g., visits with a CHW), socioeconomic stressors, several measures of parent health and well-being (resilience, stress, inflammation, immune activity, oxytocin, depression, anxiety, lifestyle, and social support) and parenting attitudes. We also collected information about child lifestyle and positive childhood experiences. Finally, we collected information on potential moderators including demographics and parent ACEs. All potential mediators and moderators were measured using well-validated tools or expert recommendations (references shown in Table S1).

- Analysis

Baseline characteristics will be assessed for both physicians and patients. In the case of physicians, we will compare physician documentation of counseling and handouts about ACEs prior to randomization to training. In the case of research participants, we will compare baseline demographics. Any differences in baseline characteristics will be adjusted for in the analysis.

The data will be analyzed using LGC modeling, which is a flexible framework for the analysis of longitudinal data that allows for better understanding of within-person and between-person variability [77]. Within the LGC framework, each individual’s repeated measurements for a given outcome (e.g., toxic stress biomarker) are modeled using a linear function that can be characterized by the intercept (which corresponds to the initial or baseline measurement), and the slope (which corresponds to the individual’s trajectory, or rate of change, over time). The average of these trajectories across individuals can be estimated, as well as the variability of trajectories. Most importantly, LGC models also allows for between-person predictors of the individual-specific intercepts and slopes.

Aim 1 will be addressed by incorporating Child-ACE scores as a predictor of the individual differences in initial status (i.e., intercept of growth trajectories). This will provide insight into whether those with higher Child-ACE scores tend to have higher toxic stress at baseline. For Aim 2, LGCs for each outcome of interest (i.e., child toxic stress biomarkers, child health and psychosocial problems) will be modeled separately. Within LGCs, the treatment indicators will be included as predictors of the slopes for the individual trajectories over time. This is equivalent to testing whether there is an interaction effect between the treatment condition and time on the outcome and will provide insight into whether individuals in the different treatment conditions tend to change over time at different rates. Within Aim 3, the impact of potential mediators of intervention efficacy will be assessed. The measurement of the potential mediating variables at the second time point (3 months) and the measurement of the outcome at the third time point (6 or 12 months) will be used. Potential moderators will also be assessed using the measurement of the outcome variables at the third or final time point, but the potential moderators will be from the baseline measurements.

In primary analyses, we will use Full Information Maximum Likelihood (FIML) to handle missing data. FIML is recommended when missing data exceeds 10% to maintain statistical power, reduce bias, and improve precision of estimates [78,79]. We will also conduct secondary analyses that do not use FIML to handle missing data to determine whether there are any substantive changes to the results. 

## 3. Results

### 3.1. Physician Changes

#### 3.1.1. Feedback from Physicians

**Figure 3:**
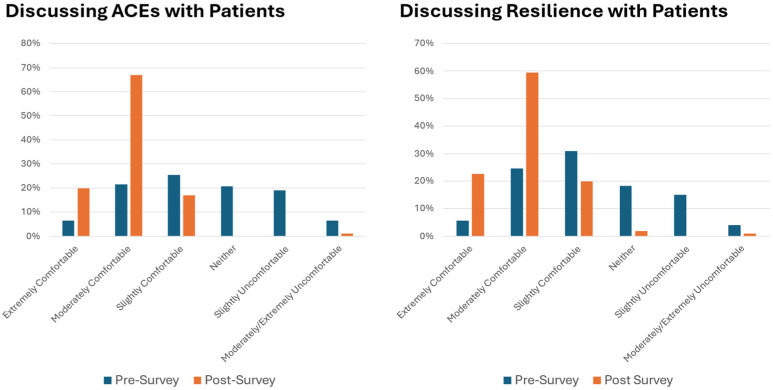
Physician self-reported comfort before and after FIRST training.

Between June 2021 and December 2024, a total of 117 resident physicians in Pediatrics or Family Medicine and a total of 27 attending physicians were trained. Based upon surveys before the training, 28% of the physicians felt moderately to extremely comfortable discussing ACEs and 30% felt moderately to extremely comfortable discussing strategies to build resilience with families. Immediately after the training, these numbers increased to 87% and 82% respectively, which was a statistically significant increase in proportions (f = 0.668, *p* < 0.0001) (Figure 3). The training also increased physician awareness of strategies to support children exposed to ACEs. Prior to the training, physicians were only familiar with referrals to community resources or behavioral health. After the training, physicians added handouts about ACEs and resilience, referrals to CHWs, and referrals to Help Me Grow for developmental concerns. In describing the strengths of the training, physicians used words such as “helpful,” “informative,” and “practical.”

In informal interviews, physicians described gaining a deeper understanding of the ways in which ACEs can influence both physical and mental health outcomes across the lifespan. They also indicated greater comfort in addressing sensitive topics related to ACEs and an increased appreciation for the role of protective factors in mitigating long-term effects. They reported improved familiarity with community and institutional resources. Educational handouts addressing ACEs, stress, and resilience were viewed as valuable, especially in situations in which families were not ready or willing to accept a referral.

#### 3.1.2. Observations of Physicians

In direct observations of well-child care visits, ACE topics and non-ACE topics were identified and discussed with equal frequency by trained and untrained physicians (Table 1). However, physicians who participated in the FIRST training were 5 times more likely to identify a concern about ACEs (10% vs. 2%) and 3 times more likely to take action (6% vs. 2%). Actions taken by untrained physicians in response to ACEs included counseling and providing community resources. Trained physicians provided counseling and community resources, but also shared handouts about ACEs and resilience, and made referrals to CHWs. For non-ACE topics, trained physicians were more likely to identify concerns (14% vs. 9%) and were marginally more likely to provide counseling (19% vs. 16%).

#### 3.1.3. Documentation by Physicians

Medical charts were reviewed for a total of 834 well-child visits. Trained providers documented counseling related to ACEs at 39% of well-child visits compared to 16% of well-child visits by untrained providers (f = 0.267, *p* < 0.001). Trained providers did not decrease counseling about lifestyle but actually documented more counseling about lifestyle (42%) compared to untrained providers (28%) (f = 0.148, *p* < 0.001). Handouts with information and resources for children exposed to ACEs were added to the after-visit summary for 16% versus 2% of well-child visits for trained and untrained providers respectively (f = 0.255, *p* < 0.001).

### 3.2. Community Referrals

#### 3.2.1. Volume and Success Rate of Referrals to Community Health Workers

At our clinic that served low-income patients who were either uninsured or had public insurance, trained providers made referrals to CHWs for approximately 10% of well-child care visits. At our clinics that served 40–60% commercial insurance patients, trained providers made referrals to CHWs for approximately 5% of well-child care visits. It was difficult to coordinate warm hand-offs to CHWs during the medical visit, so the majority of connections were made by follow-up phone calls. Approximately 34% of these referrals resulted in successful connections to CHWs.

The patient demographics of referrals by trained providers are shown in Table 2.

Referral rates mirrored the age and sex distribution of our clinic population. By race/ethnicity, Hispanic and Black patients were over-represented (referrals 68.7% and 16.8% compared to clinic populations of 44% and 10% respectively) while White and Asian patients were under-represented (referrals 10.6% and 2.8% compared to clinic populations of 18% and 14% respectively). Success rates were highest for male patients (35.7% vs. 28.5%, *p* = 0.003) and marginally higher for Black compared to White patients (35.5% vs. 27.0%, *p* = 0.05). There were no statistically significant differences in successful connections to CHWs by age or race/ethnicity.

#### 3.2.2. Risk Factors Associated with Referrals

**Figure 4:**
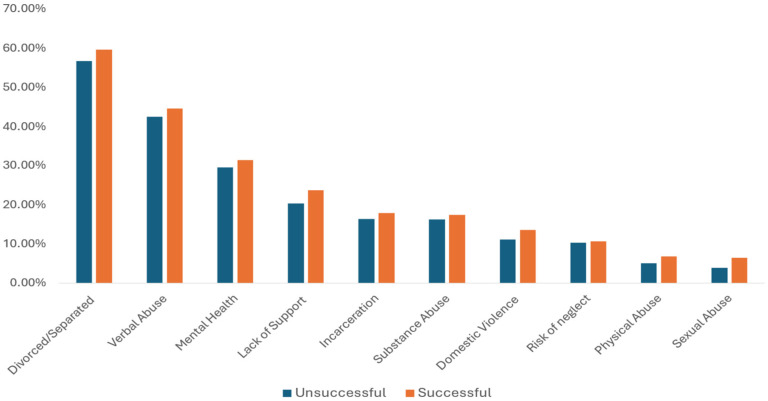
Referrals to CHWs by Specific ACEs.

Referrals were made based upon specific ACEs rather than a total ACE score but were more common for total ACE scores of 4 or more (37.5%) compared to ACE scores of 1 (24.2%), 2 (20.4%) or 3 (17.9%). Families referred to CHWs were most often challenged by parental divorce/separation, parent–child verbal abuse, and caregiver mental health problems (Figure 4). The distribution of specific ACEs in patients referred to CHWs was identical to the distribution of specific ACEs in our general patient population (i.e., divorce/separation was most frequently reported followed by verbal abuse, etc.). Approximately 9% of referrals to the Whole Family Care hub were made due to concern for ACEs only. The majority (84%) of referrals were for multiple risk factors, most commonly ACEs and socioeconomic SDOHs. A high percentage (37%) of referrals included concerns for both ACEs and developmental delay.

## 4. Discussion

The FIRST study was designed to address gaps in evidence for ACE screening [80,81]. The intervention was based upon prior research which demonstrated improvements for youth exposed to ACEs by providing parenting education, mental health counseling, linkage to social services, and social support [23]. Prior to receiving FIRST training, only about one-quarter of physicians reported feeling moderately to extremely comfortable discussing ACEs with families; after training, comfort increased to over eighty percent. These attitudinal shifts were accompanied by measurable behavioral changes. In direct observations, trained providers were significantly more likely to identify ACE-related concerns and take appropriate action when family-level concerns were present. In medical record documentation, trained providers more frequently recorded counseling related to ACEs and were more likely to provide handouts that reinforced protective factors. Importantly, these changes occurred without a reduction in counseling about lifestyle or other preventive health topics, suggesting that responses to ACEs can be integrated into routine pediatric practice without displacing other core aspects of medical care. This finding is consistent with other research showing that trauma-informed care training can improve provider knowledge, confidence, and attitudes without disrupting clinic workflow [82].

Several authors have raised concerns that screening for ACEs in primary care practice could result in harms such as patient re-traumatization, increasing patient anxiety, decreasing doctor–patient trust, and increasing stigma [83,84,85]. However, studies on implementing ACE screening in pediatric practice report high rates of acceptability among parents [83,84,85,86,87]. In our own work developing the Whole Child Assessment, parents expressed support for the importance of pediatricians knowing about family context and they did not express any concerns about ACE questions [51]. In addition, our approach to ACE screening focuses on responding to specific questions about ACEs, so concerns about mis-labeling or stigma related to an ACE score are less relevant.

Our clinical quality improvement data has important implications for implementation by other pediatric practices. While some authors have expressed concern that ACE screening would result in a high number of unnecessary referrals, we found that our referral rate was relatively low (5–10% of well-child care visits). We also found that our success in connecting families to CHWs was low (34%), although this rate was comparable to the success of other community referrals (e.g., to Help Me Grow for developmental concerns) and higher than success rates for behavioral health referrals [88,89]. While full-time onsite CHWs would improve referral success rates, this is less feasible for most pediatric practices than phone follow-up by CHWs after the pediatric visit. Thus, our approach to connecting families to additional support is a pragmatic model that other pediatric practices may be able to implement, and our future analyses of impact on health care outcomes and utilization will be relevant to these same practices.

Referral patterns by trained providers mirrored the demographics of our patient population by age and sex. However, success rates were higher for male compared to female patients. We hypothesize that this may reflect higher rates of concern for developmental delays in male patients [90]. We can test this hypothesis in future explorations of our data. We also found that our Hispanic and Black patients were disproportionately represented in referrals to CHWs. This is consistent with other literature on the high rate of SDOHs in addition to ACEs in these populations [91]. We plan to explore the associations between ACEs, SDOHs and race/ethnicity further in future analyses. Most importantly, we did not find a significant difference in successful connections to CHWs by race/ethnicity, and we actually found a trend toward a higher rate of success for Black compared to White patients, which is promising for the potential of CHW support to help reduce health disparities.

The FIRST model has the potential to be scalable across a wide range of health care settings due to the relatively low time commitment for physician and CHW training. While some resources are required to offer CHWs support, our data provides practical information to plan for resource requirements. Furthermore, our evaluation of the FIRST model includes both clinical quality improvement data which has real-world relevance about effectiveness, as well as research data that will help address critical gaps in evidence about ACEs, toxic stress and child outcomes.

Some limitations should be considered when interpreting our current quality improvement and future research data. First, our results may not be generalizable to other geographic regions or settings with different patient characteristics. Second, while our research sample has the strength of being applicable to a generally healthy pediatric patient population, it may be challenging to show improvements in health outcomes in a low-risk patient population. Future research on a high-risk patient population, such as a foster care sample, could help extend our findings. Another consideration is that our intervention is relatively low intensity with most patients in the intervention group only receiving counseling and educational handouts by trained physicians, while a smaller subset were connected to CHWs or parenting groups. Nonetheless, the FIRST intervention is comparable to the SEEK model which demonstrated favorable outcomes based upon patient and physician experience, as well as reductions in child maltreatment [92]. In addition, our research data collection includes a broad range of child and caregiver metrics which will increase our ability to observe an impact on the child, parent, or parent–child relationships.

The unique role of pediatricians in the lives of children and families provides them with the opportunity to address specific ACEs and potentially prevent toxic stress by applying what is known about protective factors. The FIRST intervention provides a pragmatic approach to addressing ACEs in the context of well-child care. Our results provide evidence that the observed physician-level changes and connections to community partners represent plausible mechanisms that could lead to improvements in stress regulation, relational health, developmental trajectories, and child health outcomes. The results of our research study will evaluate the impact of the FIRST intervention and address important questions about associations between specific ACEs, protective factors, and biomarkers of toxic stress. Future research can evaluate the application of the FIRST model in different patient populations, as well as coordination with public health and policy level changes to prevent ACEs and improve health outcomes over the lifespan.

## Figures and Tables

**Table 1:** Direct observations of well-child visits by training status.

	ACE Topics			Non-ACE Topics		
	Untrained	Trained		Untrained	Trained	
	N = 28	N = 31	*p*-Value	N = 28	N = 31	*p*-Value
Topic discussed	15%	16%	NS	26%	28%	NS
Concern noted	2%	10%	<0.0001	9%	14%	0.025
Action taken	2%	6%	0.007	16%	19%	0.102

NS = not significant.

**Table 2:** Demographics of referrals by trained providers.

	Prevalence N (%)	Success Rate %
Age		
0–2	428 (34.3%)	31.8%
3–5	303 (24.3%)	29.4%
6–8	197 (15.8%)	30.5%
9–11	149 (11.9%)	33.6%
12–17	171 (13.7%)	40.4%
Biological Sex		
Female	585 (46.9%)	28.5%
Male	663 (53.1%)	35.7%
Race/Ethnicity *		
Hispanic	819 (68.7%)	32.7%
Black/African American	200 (16.8%)	35.5%
White	126 (10.6%)	27.0%
Asian	33 (2.8%)	36.4%
Other	14 (1.2%)	28.6%

* Missing/refused for 56 patients.

## Data Availability

The data that support the findings of this study are available from the corresponding author (A.M.) due to privacy reasons.

## Supplementary Materials

The following supporting information can be downloaded at: https://www.mdpi.com/article/10.3390/children13040572/s1, Table S1: Measures collected for Building Resilient Families research participants. References [93,94,95,96,97,98,99,100,101,102,103,104,105,106,107,108,109,110,111,112,113,114] are cited in the supplementary materials.

## Abbreviations

The following abbreviations are used in this manuscript:

ACEs	Adverse Childhood Experiences
CHWs	Community Health Workers
FIRST	Families Implementing Resilient Systems Together
LGC	Latent Growth Curve
PCEs	Positive Childhood Experiences
SDOHs	Social Drivers of Health
