# Supplementary material for: Families Implementing Resilient Systems Together (FIRST)
Source: Children (Basel). 2026 Apr 20;13(4):572. doi: 10.3390/children13040572 (PMC13114711; doi:10.3390/children13040572)
Supplement: Supplementary file 1 [file children-13-00572-s001.zip › children-4217312-supplementary.pdf]

Table S1. Measures collected for Building Resilient Families research participants.

Variable	Measure(s)
Child ACEs	
Exposure, Risk	Whole Child Assessment [a,d] [51,52]; PEARLS [a] [86]
Exposure details	Structured interview about frequency, timing and modifiers of ACEs [a]; parent–child conflicts last week and full scale [b] [93]
Child Toxic Stress Biomarkers	
Biometrics	Body mass index %; resting blood pressure; mean arterial pressure; pulse; tympanic membrane temperatures bilaterally
Immune	Dried blood spot sample [94] for proinflammatory immune cell gene expression [95,96]
Inflammation	Dried blood spot sample [94] for IL-6 and CRP [97,98]
HbA1c	Hemoglobin A1c Now Self-Check for blood sugar control over 3 months
Cortisol	Hair sample for cortisol over past 3 months [99]
Aging	DNA from buccal cell samples for telomere [a,c,d] length [100]
Executive function	Minnesota executive function scale [101] and Vanderbilt inattention and hyperactivity subscales [102]
Child Health and Psychosocial Problems	
Diagnoses & utilization	Chronic diagnoses, number of urgent/ER visits and hospitalizations between baseline visit and 12-month follow-up
Development	Subscales on the Ages and Stages Questionnaire (ASQ) [103] (age 3–5 only)
Behavior	Pediatric Symptom Checklist (PSC) total score and subscales [104]
Asthma	Asthma control test total score [105] (asthma patients only)
School performance	Vanderbilt teacher school performance ratings [102]
Potential Mediators (Intervention Targets)	
Community programs	CHW contacts; number of parenting sessions attended
Socioeconomic stressors	CHW or research interview [106]
Parent resilience	Individual and family resilience [b] [18]
Parent stress & toxic stress	Perceived stress scale [107]; resting blood pressure; mean arterial pressure; pulse; passive drool for C-reactive Protein, oxytocin, and immune profile [108]
Parent mental health	Patient health questionnaire [b,c,d] [109]; generalized anxiety disorder [b,c,d] [110]
Lifestyle	Whole Child Assessment—nutrition, exercise, sleep [51,52]
Parent support	MOS social support survey [111]
Parenting	Adult Adolescent Parenting Inventory (AAPI) measures of empathy, expectations and harsh punishment [a,c] [112]
Positive childhood experiences	Questions based upon references [b] [20,113]
Potential Moderators (Covariates)	
Demographics	Birth weight, gestational age at birth, current age, biologic sex, race/ethnicity, and primary language [a]
Parent ACEs	Adult 1-page ACE tool [a,d] [4,114]

[a] Baseline visit; [b] 3-month visit; [c] 6-month visit; [d] 12-month visit.
